# Newborn clinical condition assessment using infrared thermography: correlation with the Apgar score in a prospective cohort study

**DOI:** 10.3389/fped.2025.1636667

**Published:** 2025-12-12

**Authors:** Mathilde Letouzey, Samuel Diop, Claude Elvire Pédié Kengoum, Anne Rousseau, Noémie Hot, Juliette François, Thibaud Quibel, Paul Berveiller, Pascal Boileau, François Jouen, Imen Trabelsi, Jean Bergounioux

**Affiliations:** 1 Service de Néonatalogie, Hôpital de Poissy St-Germain-en-Laye, Poissy, France; 2 Laboratoire Cognitions Humaine et Artificielle, EPHE, Université PSL, Aubervilliers, France; 3 Systèmes Intelligents en Neurologie et Réanimation Pédiatrique, R2P2, Garches, France; 4 Centre de Recherche en épidémiologie et Santé des Populations (CESP) CESP, Université Paris-Saclay, UVSQ, Inserm, UMR U1018, Montigny-le-Bretonneux, France; 5 Service de Réanimation et Neurologie Pédiatriques, Université Paris-Saclay, UVSQ, 2IC UMR 1173, AP-HP, Hôpital Raymond Poincaré, Garches, France; 6 Neurologie & Réanimation Pédiatriques, CHU Raymond Poincaré, Garches, France

**Keywords:** infrared thermography, apgar score, neonatal assessment, peripheral perfusion, newborn, thermal adaptation

## Abstract

**Objectives:**

The Apgar score remains subjective in key components despite its clinical importance. An objective method using infrared thermography could enhance neonatal assessment precision. To describe early surface thermal adaptation patterns during the first 10 min after birth using infrared thermography (IRT) and to benchmark these descriptive patterns against concurrently assigned Apgar scores. This feasibility study evaluates operational characteristics of IRT as a non-contact, objective adjunct to routine assessment; it does not establish prediction or clinical utility.

**Study design:**

Prospective cohort of 223 full-term cesarean-delivered newborns at a tertiary maternity hospital (2021–2023). Whole-body IRT was captured at 1, 3, 5, and 10 min after birth (T1–T10). Infants were described by Apgar at 1 min (≤7 vs. ≥8).

**Results:**

Descriptive visualizations showed observable differences in early surface temperature distributions for infants with lower Apgar scores at 1 min, with progressive warming and convergence between groups over time. Early rectal (central) temperatures in the low-Apgar group were sparsely recorded due to clinical priorities and are presented for transparency only.

**Conclusions:**

IRT can visualize thermal adaptation during immediate neonatal transition and may serve as a non-contact, objective adjunct to clinical assessment. Further outcome-based validation in larger, more heterogeneous cohorts is needed before clinical implementation.

**Clinical Trial Registration:** ClinicalTrials.gov, identifier NCT04483869.

## Introduction

In 1952, Virginia Apgar MD proposed a scoring system to assess newborns at 5 min after birth, based on five clinical signs: heart rate, respiratory effort, muscle tone, response to stimulation (originally termed “reflex irritability”), and color (1). Following its validation in 1958 (2), the assessment was extended to include 1-minute scoring for earlier evaluation, with the same components repeated at 1, 5, and (when indicated) 10 min.

The Apgar score comprises five components, each scored 0–2 (1): color (reflecting peripheral perfusion), heart rate, reflex irritability, muscle tone, and respiratory effort. This scoring effectively quantifies signs of neonatal depression, including cyanosis/pallor (score 0–2), bradycardia (<100 bpm = score 1, absent = 0), depressed reflexes (score 0–2), hypotonia (score 0–2), and apnea/gasping (score 0–2). Current guidelines mandate scoring at 1 and 5 min for all newborns, with continued 5 min interval assessments until 20 min for scores below 7 (3, 4). The score serves three primary functions: immediate neonatal assessment, documentation of clinical status, and (when persistently low) identification of infants who may benefit from closer monitoring. Although population-level studies have shown statistical associations between Apgar scores and long-term outcomes, the score was never intended to predict individual neurodevelopmental trajectories. Current neonatal resuscitation guidelines emphasize that only heart rate and respiratory effort (not the overall Apgar score) should guide immediate interventions. Despite its widespread adoption as the standard method for reporting neonatal status immediately after birth (5, 6), the Apgar score has notable limitations: it cannot, by itself, diagnose perinatal asphyxia or accurately predict individual neurodevelopmental outcomes (7).

Although it remains the most widely used neonatal assessment tool (8), numerous studies have identified three key limitations: inter-rater variability in subjective components (particularly color and tone assessment), confounding influences of gestational age and maternal medications, and the lack of a linear relationship with acid-base status (7, 9).

Recent systematic reviews (7, 9) have highlighted persistent gaps in the psychometric validation of the Apgar score, including the absence of an established minimal clinically important difference, inadequate statistical power for rare outcomes (such as cerebral palsy), and context-dependent predictive value. These limitations may introduce measurement bias that affects both clinical decisions and research outcomes (10, 11).

Infrared thermography, based on the measurement of infrared radiation (12), has been recently evolved for medical temperature monitoring (13–16) and diverse clinical applications, including the evaluation of inflammation and vascularization (17, 18). Abnormalities such as malignancies, inflammation, and infection cause localized increases in temperature which have been shown as hot spots (13) or as asymmetrical patterns (15) in an infrared thermogram.

The widespread adoption of this technology stems from its precision, reliability, and ease of use, offering rapid, non-contact, non-invasive, and non-radiating diagnostic capabilities for both therapeutic and preventive medical applications (19–23).

As the clinical signs captured by the Apgar score are closely related to perfusion (perfusion is the principal vector of heat distribution in the human body), we proposed to describe thermal patterns in neonates during the first minutes after birth and to benchmark these descriptive patterns against concurrently assigned Apgar scores. Specifically, we hypothesized that newborns with lower Apgar scores at 1 min would display observable differences in early surface thermal adaptation compared with higher-scoring peers.

We focused on skin surface temperature distribution rather than central temperature, as peripheral perfusion reflects the cardiovascular and respiratory components captured by the Apgar score. This proof-of-concept study aims to establish whether thermal biomarkers merit further validation against clinical outcomes.

## Methods

### Study design

This prospective cohort study investigated the use of infrared thermography for assessing neonatal clinical status immediately after birth, comparing thermal patterns with Apgar scores. The study was conducted in the Department of Gynecology and Obstetrics at Poissy-Saint-Germain General Hospital (France) between August 2020 and June 2022.

### Study population

Eligible neonates had a gestational age of 37 weeks or more, did not present any relevant malformation and were born by cesarean section (in emergency or programmed). Non-inclusion criteria were parent's refusal, maternal age less than 18 years or non-affiliation with the social security system. Cesarean-born infants were selected to standardize postnatal care (placement on radiant warmers) and minimize environmental variability. Exclusion criteria included major congenital anomalies, maternal fever >38°C, refusal of consent, or infants with unusable pictures. Neonates requiring resuscitation received immediate per-protocol care prioritized over data collection.

### Clinical and thermographic assessments

Two experienced midwives independently assigned Apgar scores at 1, 3, 5, and 10 min, in accordance with the unit's protocol for this study. The three-minute assessment was included specifically for research purposes to capture intermediate stages of thermal adaptation. Concurrently, a separate clinician acquired thermographic images at the same time points without interrupting neonatal care.

Infrared thermal imaging was conducted using a calibrated FLIR T650sc camera (640 × 480 resolution; ±1°C accuracy) positioned 1.5 meters from the radiant warmer, capturing whole-body thermograms at identical time points without disrupting neonatal care. All infants were placed under a FABIE (Mediprema) radiant warmer immediately after delivery. Warmers were initialized at a 39°C setpoint; servo-control was engaged using a skin probe affixed to the right upper quadrant once the clinical team confirmed probe adherence (typically within 1–3 min of birth). The protocol and thresholds for servo activation were identical across births per unit policy.

During thermographic acquisition, newborns remained uncovered except for a diaper to allow unobstructed visualization of the whole body surface. No covering materials were used, thereby minimizing interference with surface temperature measurements. We recorded clock times for birth, thermographic frames (T1/T3/T5/T10), and probe placement when available.

Imaging occurred under controlled ambient conditions (22–24°C; 50%–60% humidity) to minimize thermal interference.

In this study, we formed two groups: a low Apgar group, comprising newborns with an Apgar score below 8 at one minute of life, and a high Apgar group, including those with a score equal to or greater than 8.

### Image processing and analysis

**Figure 1:**
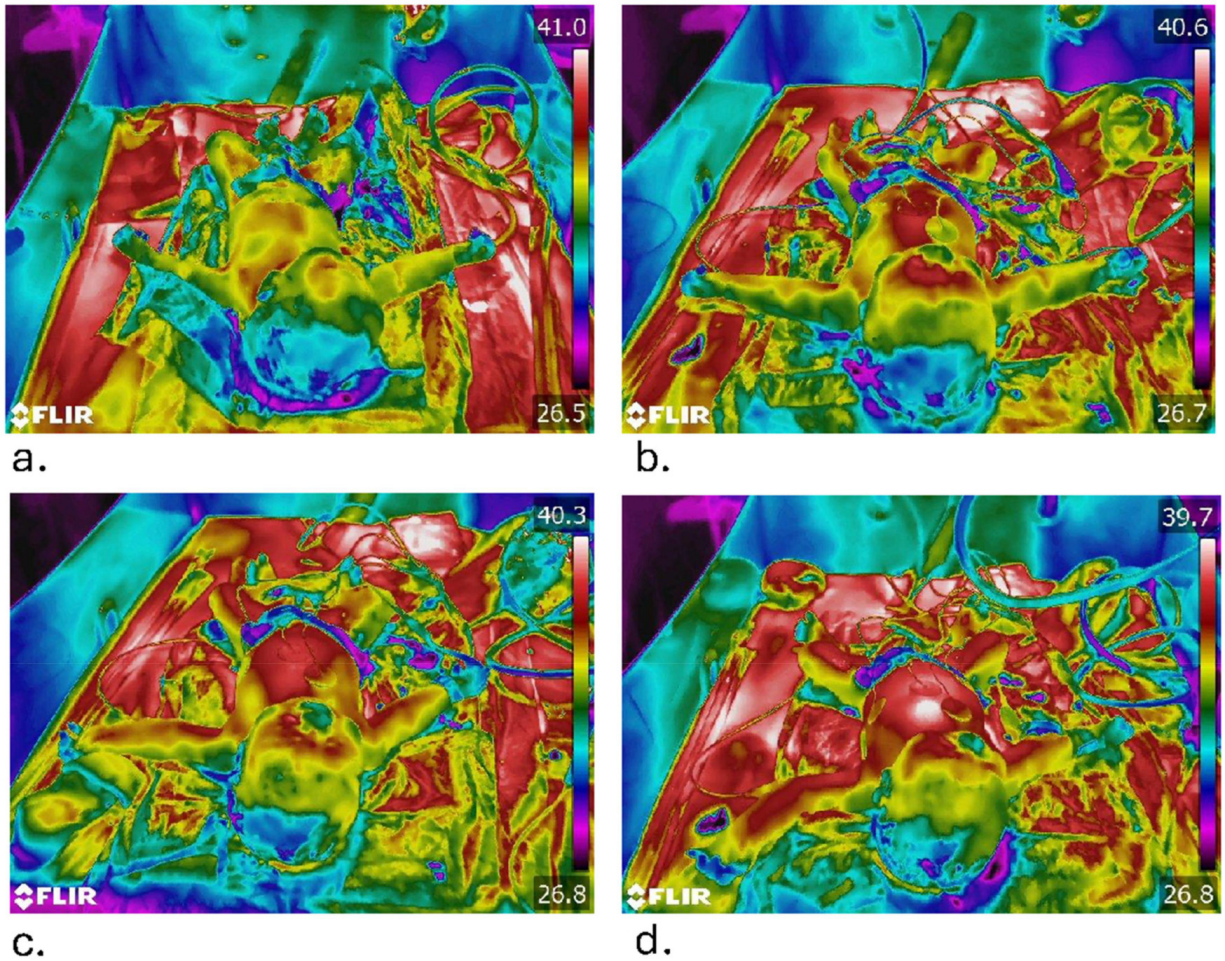
Four thermographic images of the same patient were captured at 1, 3, 5, and 10 min respectively corresponding to picture **(a–d)**, coinciding with the Apgar score assessment by two midwives. The newborn lies on the heating table, head turned towards the caregiver. We observe the evolution of temperatures over the different Apgar times, with progressive centripetal warming.

Raw thermographic data, shown in Figure 1, underwent three-stage processing using custom-developed tools: First, the Virginia software, written with Red language (François Jouen—R2P2 Laboratory) isolated neonatal anatomy through PyTorch-based PointRend segmentation combined with morphological filtering. Second, radiometric decoding via ExifTool and ImageMagick extracted pixel-level temperature values mapped to anatomical regions of interest (chest, extremities). Finally, quantitative thermal metrics were derived, including median body surface temperature and spatial thermal variability (interquartile range).

A key advantage of this automated pipeline is its low operator dependence; once the image is acquired, the entire segmentation and feature extraction process is software-driven, minimizing human interpretation bias.

### Clinical data

We collected maternal and obstetrical characteristics: maternal age, parity, maternal place of birth, body mass index, pregnancy disorders (gestational mellitus, hypertensive disorders, fetal growth restriction), cause of cesarean delivery, type of analgesia, maternal hyperthermia, and perpartum administration of antibiotics. We also collected neonatal data such as anthropometric measurements, cord blood gas, neonatal management (ventilation, intubation, chest compression), and place and purpose of transfer if adapted.

### Statistical approach

Analyses are descriptive. For thermography, we summarize body surface temperature at each time point (median and interquartile range) and display distributions with box plots. Given the group-size imbalance and missing early measurements among infants requiring urgent care, no hypothesis testing was performed. Rectal (central) temperatures are reported for transparency only at the time points where available. Baseline maternal and neonatal characteristics were compared between low-Apgar (≤7) and high-Apgar (≥8) groups using Fisher's exact test for categorical variables and Student's t-test for continuous variables. For variables with multiple categories, groups were collapsed as follows: mother's place of birth (European vs. non-European) and analgesia (neuraxial vs. general anesthesia).

All figures were generated with Python 3.12 using standard scientific libraries.

### Ethical compliance

Participation in the study was proposed to the parents of all eligible infants, who received complete written and verbal information prior to cesarean delivery in the delivery room. Parental non-opposition (opt-out consent) was documented before the initiation of any study procedure, in accordance with French ethical regulations. Neonates were enrolled at birth. The VIRGINIA study was approved by the Comité de Protection des Personnes (CPP) Île-de-France 5 (approval number 20.02.04.46311) and registered on *ClinicalTrials.gov* (NCT04483869).

## Results

Of 223 enrolled neonates, 3 were excluded due to all images being unusable (severe occlusion or blur), leaving 220 participants in the analysis. Among these, 177 (80.5%) had Apgar scores of 10 at all time points (1, 3, 5, and 10 min), and 43 (19.5%) had Apgar scores <10 at one or more time points. Based on 1 min Apgar scores, 11 infants were classified in the low Apgar group (≤7) and 209 in the high Apgar group (≥8). The number of infants per group remained constant across all time points.

Maternal and neonatal baseline characteristics are presented in Table 1. Maternal age ranged from <25 to ≥35 years (51.4% aged 25–34 years), 64.3% were born in Europe, and 58.4% had BMI ≥ 25. Gestational age was 38.5 (0.9) weeks, and birth weight was 3,315 (464) grams. Maternal and neonatal baseline characteristics are presented in Table 1. Most characteristics did not differ significantly between the low- and high-Apgar groups, including BMI, parity, gestational diabetes, fetal growth restriction, analgesia type, cesarean type, gestational age, sex, and birth weight (all *p* > 0.05). However, a significant difference in maternal age distribution was observed (*p* = 0.001), with a higher proportion of mothers aged <25 years in the low-Apgar group (27.3% vs. 1.9%). This finding should be interpreted with caution given the small absolute numbers (3 vs. 4 mothers).

**Table 1:** Maternal, obstetrical, and neonatal characteristics by Apgar at 1 min (≤7 vs. ≥8). Values are *n* (%) or mean (SD). Cesarean births only; identical radiant warmer protocol across groups. *P*-values are from comparative tests between groups.

Characteristic	Total (*N* = 220)	Apgar 1 min ≤7 (Low) (*n* = 11)	Apgar 1 min ≥8 (High) (*n* = 209)	*p*-value
Mother's age (*N* = 220)				0.001^a^
< 25 years	7 (3.2)	3 (27.3)	4 (1.9)	
25–34 years	113 (51.4)	4 (36.4)	109 (52.2)	
≥ 35 years	100 (45.4)	4 (36.4)	96 (46.0)	
BMI ≥25 (*N* = 214)	125 (58.4)	6 (54.5)	119 (58.9)	0.771^a^
Mother's place of birth^b^ (*N* = 207)				0.756^a^
European	133 (64.3)	6 (60.0)	127 (64.8)	
N-European	74 (35.7)	4 (40.0)	70 (35.2)	
Primiparous (*N* = 219)	44 (20.0)	2 (18.2)	42 (20.2)	1.000^a^
Gestational diabetes mellitus (*N* = 217)	61 (28.1)	4 (36.4)	57 (27.7)	0.516^a^
Fetal growth restriction (*N* = 218)	8 (3.7)	1 (9.1)	7 (3.4)	0.356^a^
Analgesia^c^ (*N* = 218)				1.000^a^
Neuraxial (Spinal/Epidural)	217 (99.5)	11 (100)	206 (99.5)	
General anesthesia	1 (0.5)	0	1 (0.5)	
Spontaneous labor (*N* = 220)	5 (2.3)	0	5 (2.4)	1.000^a^
Cesarean type (*N* = 212)				0.492^a^
Programmed	200 (94.3)	10 (90.9)	190 (94.5)	
Emergency	12 (5.7)	1 (9.1)	11 (5.5)	
Neonatal characteristics				
Gestational age, weeks [mean (SD)]	38.5 (0.9)	38.4 (0.9)	38.6 (0.9)	0.514^d^
Male sex	120 (54.5)	6 (54.5)	114 (54.5)	1.000^a^
Birth weight, g [mean (SD)]	3,315 (464)	3,385 (522)	3,298 (448)	0.539^d^

Footnotes for Statistical Analysis.

a Fisher's exact test.

b Categories collapsed to “European” vs. “Non-European”.

c Categories collapsed to “Neuraxial” vs. “General Anesthesia” for analysis.

d Student's *t*-test.

Clinical interventions included mask ventilation (*n* = 14), CPAP support (*n* = 38), and no infants required intubation or chest compression. Overall, 52 neonates (23.6%) required some form of resuscitation or respiratory support.

All Apgar scores were assessed by two experienced midwives independently under standardized conditions (heated resuscitation table with optimal lighting). Assessments were performed at 1, 3, 5, and 10 min post-birth.

### Central temperature analysis

**Figure 2:**
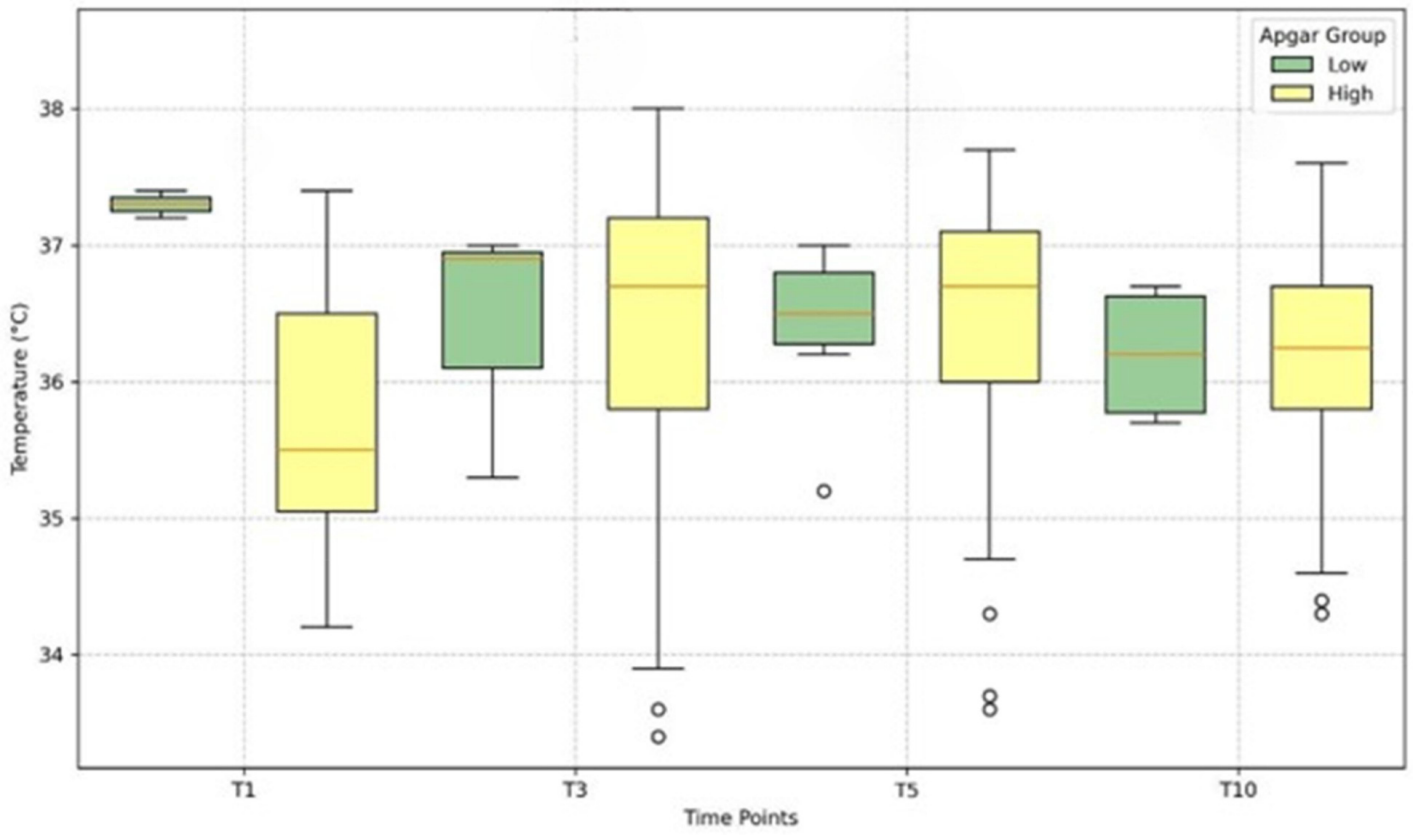
Distribution of rectal (central) temperatures by Apgar group at T1, T3, T5, T10. Box plots show medians, IQRs, and outliers. Low-Apgar (≤7) in green; high-Apgar (≥8) in yellow. Early rectal temperatures were difficult to obtain in the low-Apgar group because of clinical priorities; sample sizes are smaller at T1 and T3. Distributions are descriptive only; no hypothesis testing was performed.

Rectal temperature measurements, used as indicators of central temperature, were obtained at different time points (T1, T3, T5, and T10) for newborns categorized into low and high Apgar score groups. Figure 2 presents box plots illustrating the distribution of these central temperatures across the four postnatal time points.

For infants in the low Apgar group, early rectal temperature measurements were particularly difficult to obtain, as medical care and stabilization were prioritized during the first minutes of life. Consequently, data availability was limited at T1 and T3 for this group, and findings at these time points should be interpreted with caution.

Overall, the visual inspection of the box plots suggests that low-Apgar infants tended to have slightly higher central temperatures at T1 compared with the high-Apgar group, although the two distributions appeared more similar at later time points (T3, T5, and T10). This observation likely reflects the rapid physiological stabilization that occurs during early postnatal adaptation, rather than a persistent difference between groups.

The distributions are shown with medians and quartiles to provide a descriptive overview. No hypothesis testing was performed because of missing data and the small number of early rectal measurements in the low-Apgar group. These results are therefore descriptive only and are not intended to imply statistical significance or clinical prediction.

### Thermal profiles analysis

Obtaining skin temperature measurements, designed as thermal profile, for infants in the low Apgar group proved notably challenging for medical staff, likely due to clinical instability or procedural difficulties common in newborns requiring resuscitation or urgent care at birth. In this section, we analyze the thermal profiles derived from thermal images for two groups: newborns with low Apgar scores and those with high Apgar scores.

**Figure 3:**
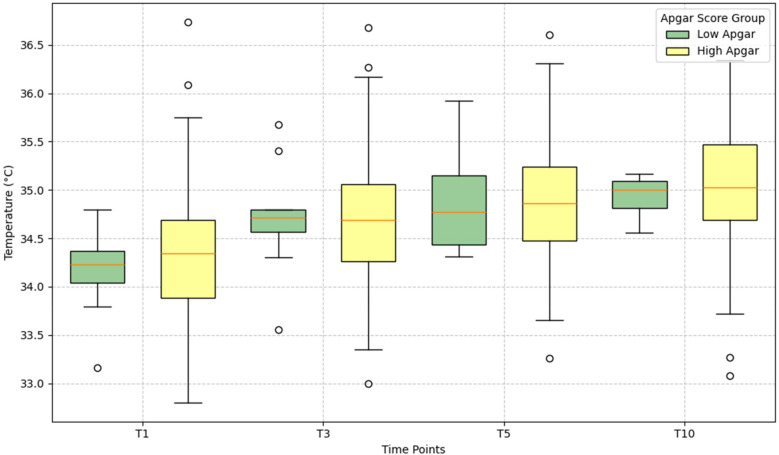
Comparison of body surface temperature distributions by Apgar group at T1, T3, T5, T10. Box plots show medians, IQRs, and outliers. Low-Apgar (≤7) in green; high-Apgar (≥8) in yellow. Both groups exhibit progressive warming with qualitative convergence by T10. Values are descriptive only; no statistical testing was applied.

The analysis focuses on body surface temperature distributions in neonates by comparing median temperatures and interquartile ranges between low and high Apgar score groups across four postnatal time points (T1, T3, T5, T10). Figure 3 illustrates this comparison using boxplots, which highlight both central tendency (median) and dispersion (IQR) while identifying individual outlying measurements. Newborns with low Apgar scores initially present with lower body surface temperatures than their high-scoring counterparts. This temperature graph tracks both groups across four points, revealing a consistent warming pattern. While the low Apgar group shows a gradual temperature increase with minimal variation, the high Apgar group displays greater temperature variability throughout. By the final measurement (T10), both groups converge toward similar median temperatures, though the high Apgar cohort maintains wider temperature ranges. This visualization effectively captures how initial physiological differences between these newborn groups gradually diminish as their thermoregulatory systems stabilize over time. Distributions are presented with medians and quartiles in box plots; no hypothesis testing was performed due to group-size imbalance and missing early measurements in the low-Apgar group. All findings should be interpreted as descriptive patterns rather than inferential results.

### Visualization of temperature-class distribution

**Figure 4:**
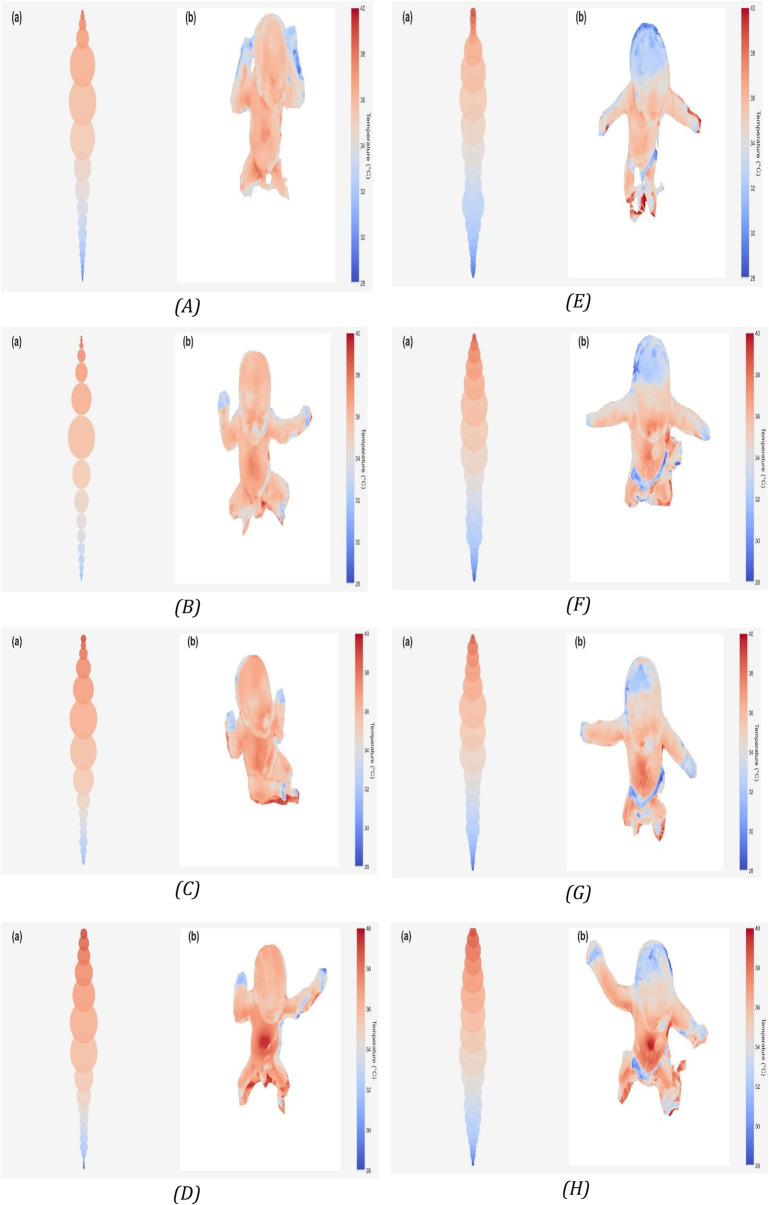
Visualization of temperature-class distribution and spatial thermal patterns in two representative newborns. Panels **(A–D)** correspond to patient 110 (low Apgar ≤ 7); panels **(E–H)** to patient 163 (high Apgar ≥ 8). Each time point (T1, T3, T5, T10) includes a bubble plot (left) showing the proportion of pixels in each temperature range and the corresponding thermographic image (right). Bubble size reflects the relative surface area of each temperature class; color corresponds to temperature level on a shared scale across all panels.

Figure 4 illustrates the evolution of surface temperature patterns for two representative newborns, one from the low-Apgar group (patient 110, Apgar ≤ 7) and one from the high-Apgar group (patient 163, Apgar ≥ 8), at four postnatal time points (T1, T3, T5, T10).

Each time point includes two complementary views: (1) Left panel: a bubble plot summarizing the proportion of body surface pixels within each temperature range. Bubble size is proportional to the relative area represented by that temperature range, and color corresponds to temperature level on the shared scale. (2) Right panel: the corresponding infrared thermogram showing the spatial distribution of temperatures across the newborn's body.

Together, these paired visualizations provide both a quantitative overview of temperature-class composition and a spatial map of heat distribution.

Qualitatively, both infants show a progressive shift from cooler to warmer temperature ranges over time, reflecting postnatal thermal adaptation. In the low-Apgar infant (patient 110), cooler extremities persist longer, and warming occurs more gradually. In contrast, the high-Apgar infant (patient 163) demonstrates faster, more homogeneous warming and earlier stabilization. By T10, both infants reach a relatively uniform thermal distribution, consistent with effective thermoregulation.

These visual representations are intended to illustrate the temporal dynamics of thermal adaptation rather than provide quantitative measurement or inference. The temperature ranges are displayed on an identical color scale for all panels to enable visual comparison. No statistical analysis was applied; findings are descriptive only.

Individual surface temperature trajectories for two representative newborns (patient 110, low Apgar ≤ 7; patient 163, high Apgar ≥ 8) measured at T1, T3, T5, and T10. Data illustrate the descriptive trend of gradual warming and temperature stabilization during early postnatal adaptation.

## Discussion

“Birth is the riskiest moment of your life. It's fundamental to quickly assess a newborn's status and immediately identify those who need urgent care.” (V. Apgar 1953). Birth is still a high-risk event in 2025: a newborn is 500 times more likely to die at birth than during the rest of their life. Assessing the newborn's *clinical condition* is a fundamental step, as it determines both the immediate management and the long-term prognosis.

The Apgar score, introduced by Virginia Apgar, has played a pivotal role in standardizing this early evaluation. Its simplicity and reproducibility have made it the global reference for assessing neonatal vitality. However, inter-observer variability and the subjectivity of some components, particularly color and tone, remain important limitations. The present study explored whether infrared thermography, a non-contact and objective technique, could provide complementary information about early adaptation by visualizing peripheral perfusion patterns immediately after birth.

Humans are homeothermic beings, and thermoregulation is a key physiological process, particularly during the neonatal transition. Heat is generated through non-shivering thermogenesis in brown adipose tissue and transported by blood flow, making temperature distribution an indirect marker of circulatory adaptation (25–28) and a key aspect of neonatal physiology (24). In this context, surface thermography offers a way to monitor peripheral heat diffusion, which is closely linked to perfusion and vascular regulation in the minutes following delivery.

In this feasibility study, infrared imaging revealed observable differences in surface temperature distribution between newborns with low and high Apgar scores during the first minutes of life. While rectal (central) temperature measurements suggested a tendency toward higher initial values in low-Apgar infants, likely related to delayed cooling, surface thermal profiles demonstrated a progressive warming pattern and eventual convergence between groups. These findings are descriptive and should be interpreted qualitatively, but they suggest that infrared thermography may capture subtle differences in thermal adaptation that parallel circulatory stabilization.

The visual representation developed for this study (Figure 4) was designed as a straightforward graphical tool for summarizing thermal data. By simultaneously displaying the spatial and quantitative distribution of temperature ranges, this visualization provides a rapid, intuitive overview of each newborn's thermal adaptation pattern. Such representations could, in future work, support automated or semi-automated analyses for early assessment of peripheral perfusion.

The immediate potential of thermal imaging lies in its ability to serve as a non-contact, objective adjunct to conventional clinical assessment. In practice, a thermal camera could be mounted above the resuscitation table to provide real-time, automated visual feedback on perfusion and thermal adaptation without disturbing care. Future work should focus on developing and validating machine-learning algorithms capable of recognizing characteristic thermal patterns over time. Such approaches could eventually generate a continuous “thermal adaptation score” to complement, rather than replace, the Apgar score. Larger prospective studies will be required to evaluate whether these thermal biomarkers correlate with short-term morbidities such as prolonged respiratory support or early signs of hypoxic-ischemic injury.

The broader variability observed among high-Apgar newborns likely reflects normal physiological diversity in thermal adaptation rather than a technical artifact. In contrast, the relative homogeneity in the low-Apgar group may arise from both smaller sample size and reduced thermal reactivity during early circulatory compromise. Importantly, these descriptive differences do not imply that infrared thermography can increase the reliability of the Apgar score. Rather, thermography provides an objective, quantitative view of one physiological component—peripheral perfusion—without assessing respiratory effort, heart rate, or reflex response. The purpose of thermal imaging at this stage is not to predict the Apgar score, but to explore whether this technology can eventually serve as a non-contact adjunct to standard assessment by quantifying aspects of neonatal adaptation that are otherwise evaluated qualitatively.

This study has several limitations. In addition to technical constraints, methodological factors may have influenced our observations. Maternal temperature was not systematically documented, and subtle variations could have affected early neonatal thermal readings despite exclusion of febrile mothers. All thermographic measurements were performed under a standardized radiant warmer, which may have impacted absolute skin temperature values but was identical across participants. Missing data were more frequent in the low-Apgar group due to clinical priorities during resuscitation, limiting early comparisons. Finally, restricting inclusion to term cesarean deliveries enhanced environmental control but reduced generalizability to vaginal or preterm births. Future studies should include more diverse delivery contexts and systematically record maternal and environmental parameters to strengthen interpretability.

Furthermore, although our cohort included infants with diverse skin tones, this factor is unlikely to have influenced thermal measurements. In long-wave infrared thermography, human skin emissivity is uniformly high across all pigmentation levels (ε ≈ 0.97–0.98), resulting in minimal bias related to skin color (13, 15). Additionally, the significant difference in maternal age distribution between groups, with younger mothers overrepresented in the low-Apgar group, represents a potential confounding factor. However, the small sample size of the low-Apgar group (*n* = 11) precludes adjustment for this variable or definitive conclusions about its influence on the observed thermal patterns.

In summary, this proof-of-concept study supports the feasibility of infrared thermography for observing neonatal thermal adaptation immediately after birth. While additional validation in larger and more heterogeneous cohorts is necessary, these findings provide a foundation for developing automated, quantitative tools that could enhance the objectivity and precision of early neonatal assessment.

## Conclusion

This proof-of-concept study demonstrates the feasibility of infrared thermography for visualizing thermal adaptation patterns in the first minutes of life. The descriptive differences observed between infants with low and high Apgar scores suggest that thermography could serve as a non-contact, objective adjunct to conventional assessment. Future work will refine image-processing methods and develop automated algorithms to analyze thermal dynamics in real time. Validation in larger, more heterogeneous cohorts will be essential to determine clinical relevance and generalizability. This study provides a foundational framework for using thermal imaging as a complementary, non-invasive tool to monitor the physiological transition at birth.

## Data Availability

The datasets presented in this article are not readily available because Patient Privacy: Contains sensitive neonatal clinical data. Full thermographic images may reveal identifiable features. Requests to access the datasets should be directed to imen.trabelsi@ephe.psl.eu.
