# Supplementary material for: Newborn clinical condition assessment using infrared thermography: correlation with the Apgar score in a prospective cohort study
Source: Front Pediatr. 2025 Dec 12;13:1636667. doi: 10.3389/fped.2025.1636667 (PMC12741121; doi:10.3389/fped.2025.1636667)
Supplement: Supplementary file 1 [file Supplementaryfile1.docx]

Figure S1 : Flowchart of neonatal participant selection and exclusion.
