# Supplementary material for: Newborn clinical condition assessment using infrared thermography: correlation with the Apgar score in a prospective cohort study
Source: Front Pediatr. 2025 Dec 12;13:1636667. doi: 10.3389/fped.2025.1636667 (PMC12741121; doi:10.3389/fped.2025.1636667)
Supplement: Supplementary file 2 [file Supplementaryfile2.docx]

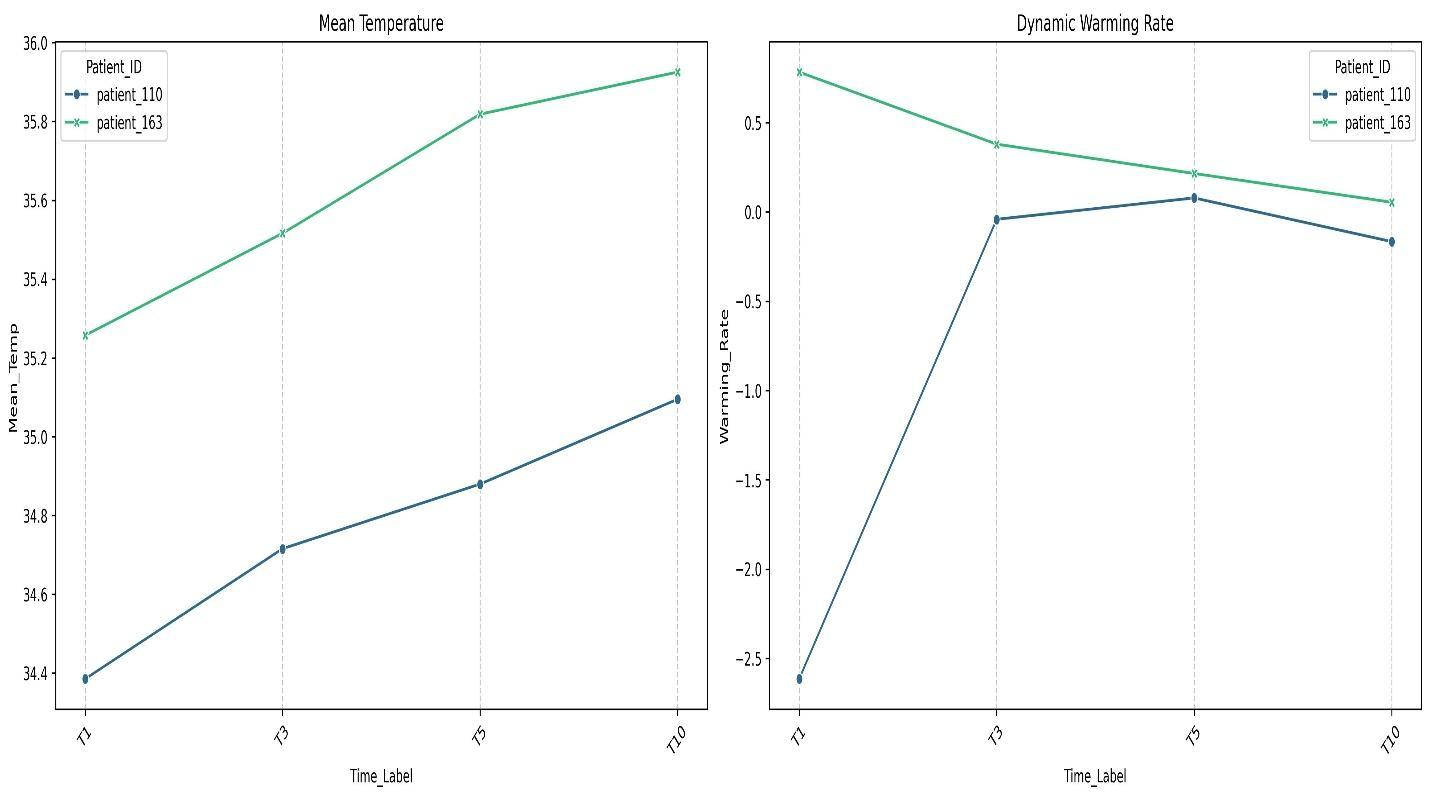
 *Figure S2. Individual surface temperature trajectories for two representative newborns (patient 110, low Apgar ≤ 7; patient 163, high Apgar ≥ 8) measured at T1, T3, T5, and T10. Data illustrate the descriptive trend of gradual warming and temperature stabilization during early postnatal adaptation.*
